# Proposal of a novel protocol using estimated cardiac index fractional dose to improve aortic contrast enhancement for early-phase dynamic CT

**DOI:** 10.1097/MD.0000000000029410

**Published:** 2022-06-24

**Authors:** Tadashi Kuba, Akihiro Tokushige, Sadayuki Murayama, Shinichiro Ueda

**Affiliations:** a Department of Clinical Research and Quality Management, Graduate School of Medical Science, University of the Ryukyus, Okinawa, Japan; b Department of Clinical Pharmacology and Therapeutics, Graduate School of Medical Science, University of the Ryukyus, Okinawa, Japan; c Department of Radiology, Graduate School of Medical Science, University of the Ryukyus, Okinawa, Japan.

**Keywords:** 3D-CTA, age, aortic contrast enhancement, bolus tracking, cardiac index, computed tomography, contrast medium, fractional dose

## Abstract

Maximum aortic computed tomography value (CTV) is difficult to control because of variations in cardiac function and patient physique. Therefore, to improve early-phase aortic enhancement on dynamic computed tomography (CT), we developed an estimated cardiac index fractional dose (eciFD). The eciFD protocol is a novel and original protocol for administering fractional dose (FD), representing the amount of iodine per unit body weight per injection duration, based on cardiac index (cardiac output divided by body surface area) as estimated by age in early-phase dynamic CT. At the time of administration, by selecting FD based on the patient's age and selecting a parameter that can achieve this FD, an aortic CTV ≥300 HU (ACTV≥300) can be obtained. This study aimed to investigate aortic enhancement on CT angiography using the eciFD protocol.

This retrospective study investigated 291 consecutive patients who underwent dynamic CT from neck to abdomen after recommendation of the eciFD protocol at our institution. We compared early-phase aortic CTV distributions by scan delay between an eciFD group (eciFD applied, n = 135) and a non-eciFD group (eciFD not applied, n = 80). The effect of eciFD on early-phase ACTV≥300 was evaluated using logistic regression analysis adjusted for several potentially meaningful clinical confounders related to aortic CTV, namely male sex, heart rate ≤80 beats/min, estimated glomerular filtration rate ≤40 mL/min, use of eciFD, bolus tracking (BT), history of myocardial infarction, and order from the emergency center.

The eciFD protocol was a significant factor for early-phase ACTV≥300 after adjusting for several confounders (odds ratio 3.03; 95% confidence intervals 1.59–5.77; *P* = .001). No interaction was seen between BT and eciFD protocol (p for interaction = 0.76). In terms of CTV distribution, with both a fixed scan delay time and BT, the eciFD group showed a high aortic CTV. The combination of eciFD protocol with BT provided a particularly high percentage of patients with ACTV≥300 (86.4%).

The eciFD protocol was useful for improving aortic contrast enhancement. These findings need to be validated in a randomized controlled study.

## 1 Introduction

In our institution, dynamic computed tomography (CT) of the cervico-pelvic region is often obtained for cases requiring a trauma pan scan, investigation of aortic aneurysm or dissection, or in preoperative workup for malignancy. Many such cases require 3-dimensional (3D) computed tomography angiography (CTA), so our target computed tomography value (CTV) for the aorta in the early phase is 300 to 350 HU. This target comes from a study by Higashigaito and Schmid^[1]^ on the volume of contrast required for different tube voltages in 3D-CTA, when the target value for aortic CTV was 300 to 350 HU.

In our dynamic CT protocol, a contrast medium (CM) dose of 600 mgI/kg body weight is injected for 30 seconds. While bolus tracking (BT) is usually used to determine the scan delay, aortic CTV remains difficult to control, and the above-mentioned target is often not reached.

Regarding CT of the heart, Matsumoto et al^[3]^ reported the possibility of entering data for the weight, height, and cardiac output (CO) of the patient into a contrast enhancement optimizer they had developed to yield the target CTV from the displayed imaging protocol, thus optimizing imaging effects for each patient. However, such devices need CO as measured from cardiovascular monitoring, and such data are commonly unavailable.

To more easily achieve the same purpose, we developed a CM injection protocol that takes into account the fractional dose (FD), injection duration, body weight (see Equation (1) below) and cardiac index (CI), as the CO divided by the body surface area (BSA).

Use of a FD is considered to improve the reproducibility of the time enhancement curve (TEC), as the curve showing the CTV of the aorta at each time point after injection of the CM. This improvement is achieved by keeping the injection duration and amount of iodine per unit body weight constant. FD can obtain stable contrast enhancement according to the characteristics of the individual patient.^[4]^ In particular, this method is often used in CT studies of the coronary arteries. However, the variability of cardiac function among patients affects the reproducibility of the TEC, and maximum aortic CTV remains difficult to control.^[5,6]^

We therefore hypothesized that provision of the FD in proportion to the CI by age strata would offer a more stable and reproducible TEC, facilitating control of aortic CTV. We named this new protocol the “estimated cardiac index fractional dose” (eciFD) protocol.


(1) FD=dose of iodine per unit body weight(mgI/kg)/injection duration(s)

The purpose of this study was to investigate whether the eciFD protocol contributes to improved early-phase enhancement of the aorta on dynamic CT from the neck to the thoracoabdominal region.

## 2 Methods and materials

### 2.1 Ethics statement

The institutional review board at our institution approved the study protocol (approval no. 2017-53-2). Because of the retrospective nature of this study and the de-identification of personal data, the board waived the need for informed consent. The present study was conducted in accordance with the Declaration of Helsinki^[7]^ and the regulations of the Japanese Ministry of Health, Labour and Welfare.

### 2.2 Study design and subjects

After deciding to recommend the eciFD protocol at our institution, a total of 304 patients underwent dynamic CT from the neck to the abdomen with 1-step injection of CM between October 1, 2017 and October 31, 2018. Among these, patients with placement of a stent in the thoracic or abdominal aorta, presence of aortic dissection or aneurysm, ruptured aortic aneurysm, Marfan syndrome, or performance of contrast studies using a Bard PowerPicc (C.R. Bird, Salt Lake City, UT) were excluded.

**Figure 1:**
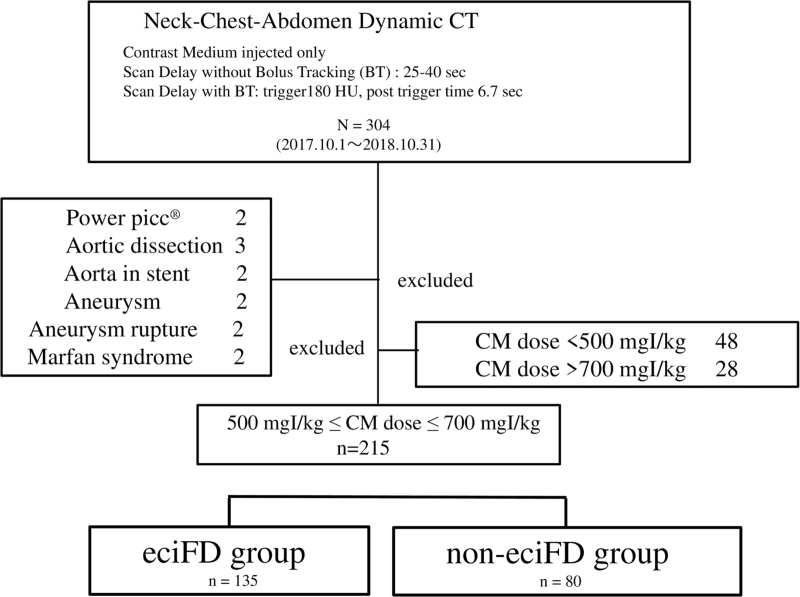
Flow diagram for inclusion of patients. Between October 1, 2017 and October 31, 2018, a total of 304 patients underwent dynamic CT from the neck to the abdomen with 1-step injection of CM. Among these, patients with placement of a stent in the thoracic or abdominal aorta, presence of aortic dissection or aneurysm, ruptured aortic aneurysm, Marfan syndrome, or performance of contrast studies using a Bard Power Picc (C.R. Bird, Salt Lake City, UT) were excluded. Cases in which the eciFD protocol was obviously infeasible, or the dose calculated in accordance with the eciFD protocol was significantly mismatched to body weight, or the CM dose was <500 mgI/kg or >700 mgI/kg were also excluded. A final total of 215 patients was included in the study. CM = contrast medium, CT = computed tomography, eciFD = estimated cardiac index fractional dose.

Cases in which the eciFD protocol was obviously infeasible, or the dose calculated in accordance with the eciFD protocol was significantly mismatched to body weight, or the CM dose was <500 mgI/kg or >700 mgI/kg were excluded. A final total of 215 patients was included in the study (Fig. 1).

We obtained demographic information for all patients, including age, height, weight, body mass index (BMI), BSA, lean body mass (LBM), CI, CO, heart rate (HR), systolic blood pressure (sBP), diastolic blood pressure (dBP), and estimated glomerular filtration rate (eGFR). Values obtained for height, weight, BP, HR, and eGFR were those recorded closest to the time of CT.

Patient comorbidities defined as cerebral infarction (non-cardiogenic), myocardial infarction (MI), heart failure (HF), coronary revascularization, valvular disease, pulmonary arterial hypertension, diabetes mellitus (DM), hypertension (HT), hyperlipidemia (HL), peripheral arterial disease, and liver cirrhosis were recorded from the medical history in the electronic medical record. Coronary revascularization was defined as a history of either coronary artery bypass grafting or percutaneous coronary intervention, and valvular disease was defined as aortic valve replacement or mitral valve replacement. In addition, cases in which a scan request was received from the emergency center (EC) were collected as an order from the EC.

**Figure 2:**
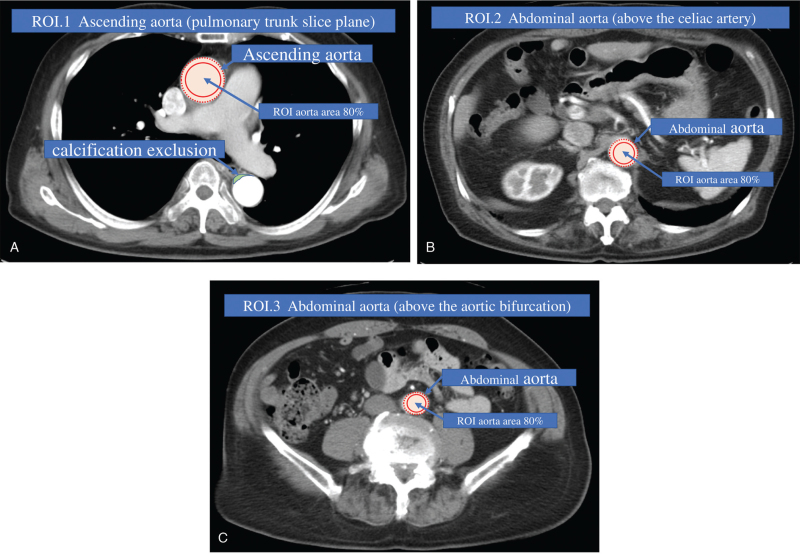
Sites for regions of interest. Aortic CTV is defined as the average of the value taken from 3 aorta sites: at the level of the pulmonary artery bifurcation; just above the level of the celiac artery; and at the level of the bifurcation of the common iliac artery. Region of interest (ROI) in measurement is defined as 80% of the arterial area. CTV = computed tomography value.

We obtained contrast-related information that included early-phase aortic CTV, intravenous injection of contrast into the left upper extremity or not, CM dose in milligrams of iodine per kilogram body weight, FD in milligrams of iodine per kilogram body weight per second, injection duration, scan delay, and BT from electronic medical records and the Picture Archiving and Communication System (PACS) archive. Aortic CTV is defined as the average of values from 3 aorta sites: at the level of the pulmonary artery bifurcation; just above the level of the celiac artery; and at the level of the aortic bifurcation (Fig. 2).

### 2.3 CT equipment and materials

Imaging data were acquired using a Discovery750HD and a Light speed VCT64-row multidetector-row CT scanner (GE Healthcare Japan, Tokyo, Japan). For the use of CM, the following equipment and materials were used: contrast injector, DUAL SHOT GX (NemotoKyourindou, Tokyo, Japan); extension tube, Nipro Extension tube (NiproCorp., Osaka, Japan); and needle for intravenous injection, Share shield II 20G (Terumo Corporation, Tokyo, Japan). For CM, a 50- or 100-mL syringe of 300- or 370-mgI/mL Oypalomin (Fuji Pharma Co., Tokyo, Japan), 100-mL syringe of 300- or 350-mgI/mL Ioverin (Teva Takeda Pharma, Aichi, Japan), or 150-mL syringe of 300-mgI/mL Omnipaque (Daiichi Sankyo, Tokyo, Japan) warmed to 37°C was used. Image analysis was performed using PACS (AstroStage, Tokyo, Japan).

### 2.4 CT scanning methods

Scanning parameters for CT were: tube voltage, 120 kV; tube current, automatic; noise index, 11.0 to 12.0; rotation speed, 0.5 sec/rotation; helical pitch, 1.375 (1/rot); and reconstruction kernel, ‘std’. CT images were 5.0 mm thick with an image interval using an adaptive statistical iterative reconstruction of 50%, with the field of view adapted to the individual physique of the patient.

### 2.5 Contrast-enhanced CT and eciFD

The eciFD protocol was created based on data from the early-phase aortic CTV distribution map from January to December 2015, including 298 dynamic CT scans from the neck to the abdomen, a scan delay of 35 seconds, and an injection duration of 30 seconds.

The 60- to 69-year-old age group with aortic CTV 300 to 350 HU included 18 cases, with a median aortic CTV of 321 HU (range, 310–328 HU), a median age of 65 years (range, 62–67 years), and a median FD of 17.9 mgI/kg/sec (range, 16.9–19.3mgI/kg/sec).

As a result, obtaining an aortic CTV of 300 to 350 HU for individuals in their 60s would require an FD of about 18 mgI/kg/sec, and according to “Physiology of Guyton and Hall”^[11]^ the CI for individuals in their 60s was 2.6 L/min/m^2^.

From these values, FD for each age was calculated using the following Equation (2):


(2) Patient eciFD=18×[CI for patient age]/2.6

The CI for each age group was as follows from “Physiology of Guyton and Hall”^[11]^: CI for teenagers, 4.5 L/min/m^2^; CI for individuals in their 20s, 4.0 L/min/m^2^; CI for individuals in their 30s, 3.5 L/min/m^2^; CI for individuals in their 40s, 3.1 L/min/m^2^; CI for individuals in their 50s, 2.9 L/min/m^2^; and CI for individuals in their 70s, 2.5 L/min/m^2^. These values were entered into Equation (2), resulting in an eciFD for each age (Table 1). The procedure for setting the amount of CM to be injected, injection duration, and injection rate to achieve eciFD is described. However, iodine content per body weight was approximately 600 mgI/kg.

**Table 1:** Estimated cardiac index FD protocol.

Age	0–10s	20s	30s	40s	50s	60s	70s-
CI (L/min/m^2^)	4.5	4.0	3.5	3.1	2.9	2.6	2.5
Patient's eciFD (mgI/kg/sec)	31	28	24	21	20	18	17

10s = teens, 20s = twenties, 30s = thirties, 40s = forties, 50s = fifties, 60s = sixties, 70s = seventies, CI = cardiac index, eciFD = estimated cardiac index fractional dose, FD = fractional dose.

The amount of CM to be injected was calculated using Equation (3):


(3) CM volume(mL)=[amount of iodine per body weight(mgI/kg)×patient weight]/amount of iodine per unit CM(mgI)

Injection duration was calculated by dividing the amount of iodine per body weight from Equation (1) using the eciFD appropriate for the age of the patient (Table 1). Injection rate was calculated by dividing the amount of CM injected by the injection duration. The maximum injection rate was 6.0 mL/sec.

**Figure 3:**
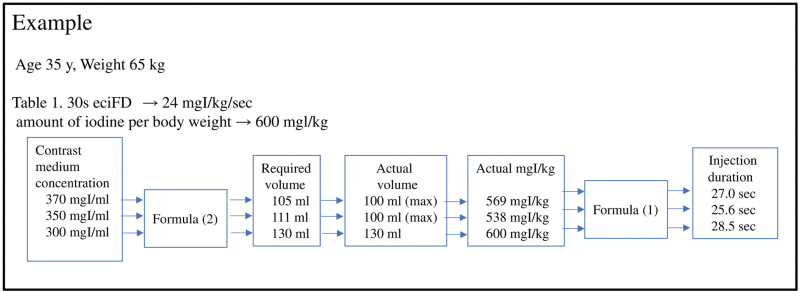
Example. For a 35-year-old individual weighing 65 kg, the eciFD for 30s is 24 mgI/kg/sec from Table 1. When the amount of iodine per body weight is 600 mgI/kg, 3 concentrations of CM can be used: 370 mgI/mL, 350 mgI/mL, or 300mgI/mL; and the required volumes for each of these are 105 mL, 111 mL, and 130 mL according to Equation (2). However, the actual volumes are 100 mL, 100 mL, and 130 mL due to the standardization of CM. The actual iodine dose per body weight is therefore 569 mgI/kg, 538 mgI/kg, or 600 mgI/kg, with injection durations of 27 seconds, 25.6 seconds, or 28.5 seconds according to Equation (1). Figures 4 and 5 were created by calculating each body weight and each age using the same procedure described above. When administering CM, the concentration, volume, and injection duration of the CM are determined based on the age and weight of the patient to support CM administrators. CM = contrast medium, eciFD = estimated cardiac index fractional dose.

**Figure 4:**
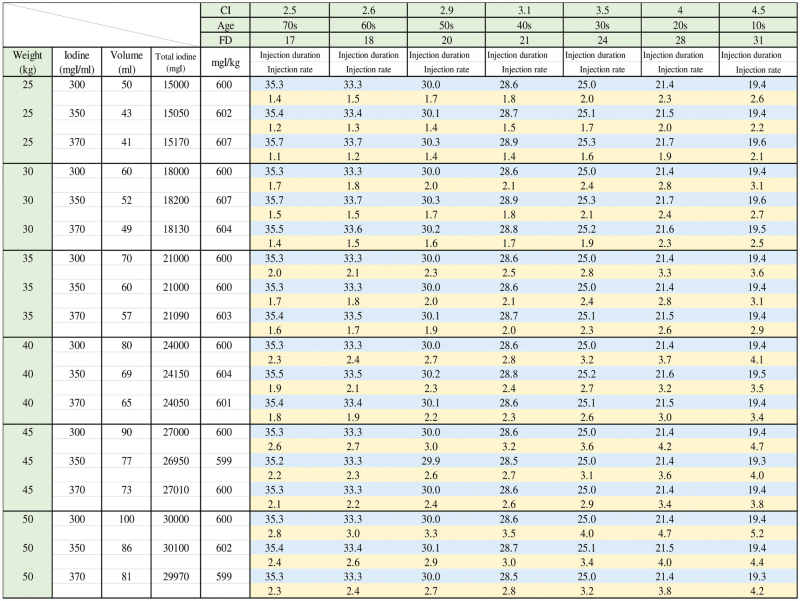
FD for each CI and age, and parameters for achieving FD (i.e., iodine dose per body weight, dosage, injection duration, and injection rate) for each available CM concentration in the range of 25 to 50 kg (at 5-kg intervals) to support CM administrators. CM = contrast medium, CI = cardiac index, FD = fractional dose.

**Figure 5:**
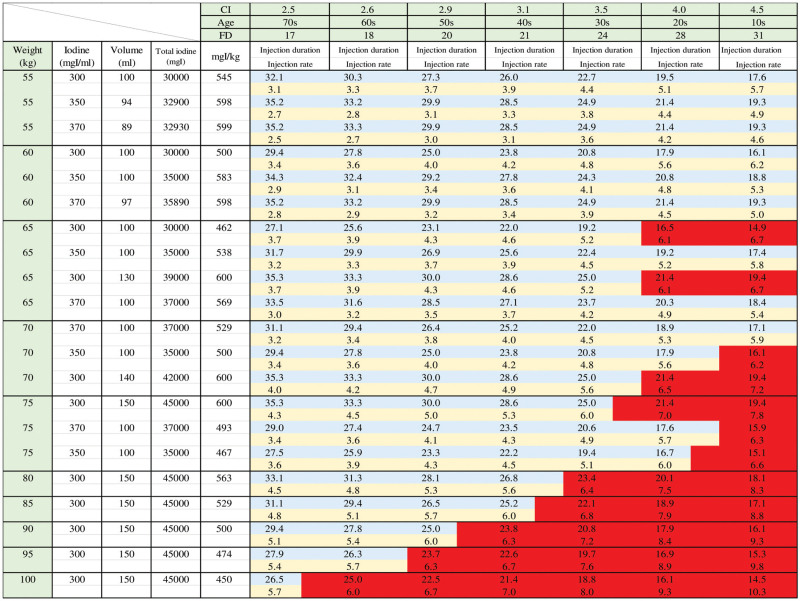
FD for each CI and age, and parameters for achieving FD (i.e., iodine dose per body weight, dosage, injection duration, and injection rate) for each available CM concentration in the range of 55 to 100 kg (at 5-kg intervals) to support CM administrators. Red area means that the maximum injection rate of 6.0 mL/sec is exceeded and is not a usable parameter. CM = contrast medium, CI = cardiac index, FD = fractional dose.

Simple control charts are shown in Figures 4 and 5. These were created using the procedure in Figure 3. Such charts are meant to assist the CT scan personnel. The injection parameters used to achieve the FD indicated by eciFD can be obtained from the age and weight of the individual patient. However, the amount of CM and the injection rate can also be obtained by automatic calculation by the CM automated injector, and therefore were accepted as the injection protocol in this study. These parameters were obtained by entering the FD, CM concentration, and injection duration into the CM automated injector (DUAL SHOT GX; NemotoKyorindo, Tokyo, Japan).

The group scanned without the BT technique was defined as the fixed scan delay (FIX) group, with scan delay set at 25 to 40 seconds. The BT technique was recommended if the injection duration was ≤30 seconds. The group scanned using the BT technique was defined as the BT group, the region of interest was placed just above the celiac axis, and when the aortic CTV exceeded 180 HU, diagnostic CT was initiated after 6.7 seconds.

### 2.6 Definitions

BSA, LBM, and CO were calculated according to the following formulae (Equations 4–7).

BSA was estimated as follows:


(4) $$\text{BSA}({\text{m}}^{2})=\text{heigh}{\text{t}}^{0.725}\;\times \text{weigh}{\text{t}}^{0.425}\times 0.7184$$

where weight is measured in kilograms and height is measured in meters.

LBM was estimated as follows:


(5) $$\text{Male LBM}(\text{kg})=(1.1\times \text{weight})-128[\text{weigh}{\text{t}}^{2}/{\left(100\times \text{height}\right)}^{2}]$$

(6) $$\text{Female LBM}(\text{kg})=(1.07\times \text{weight})-148[(\text{weigh}{\text{t}}^{2}/{\left(100\times \text{height}\right)}^{2}]$$

where weight is measured in kilograms and height is measured in meters.

CO was estimated as follows:


(7) CO(L/min)=CI×BSA

### 2.7 Evaluation

The eciFD group was defined as the group receiving the FD or higher according to the eciFD protocol specified in Table 1, and the non-eciFD group was defined as the group administered less than the FD of the eciFD protocol.

First, we compared the eciFD group with the non-eciFD group and evaluated the aortic CTV, and the percentage of patients with aortic CTV ≥300 HU (ACTV≥300).

Second, the effect of eciFD on early-phase ACTV≥300 was evaluated using logistic regression analysis adjusted for several potentially meaningful clinical confounders related to aortic CTV (male, HR ≤80 beats/min, eGFR ≤40 mL/min, BT, MI, and order from the EC). Age and weight were quite different between eciFD and non-eciFD. However, these factors could not be included as adjustment factors because multicollinearity is a concern since these factors are so strongly associated with eciFD, as a protocol in which the patient is administered a FD proportional to the predicted CI of the age group. Third, ACTV≥300 was evaluated for FIX/BT status in the eciFD and non-eciFD groups.

### 2.8 Statistical analyses

Descriptive statistics are presented as the frequency (percentage) for categorical variables and mean ± standard deviation or median (interquartile range) for continuous variables. Data were compared using the chi-square or Fisher exact test for categorical variables, a 2-sample *t*-test for a normal distribution, or the Wilcoxon rank-sum test for non-normal distributions of continuous variables. Multiple logistic regression modeling was used to identify factors associated with ACTV≥300, expressed as odds ratios (ORs) with 95% confidence intervals (CIs). Continuous variables were dealt with as binary variables categorized by clinically meaningful boundaries. All analyses were conducted using JMP version 15.0 (SAS Institute, Cary, NC). All statistical analyses were 2-tailed, with values of *P* < .05 considered statistically significant.

## 3 Results

### 3.1 Baseline characteristics for all patients

Baseline characteristics of all patients enrolled in this study are shown in Table 2. Median age was 67 years, and 127 patients (59.1%) were male. Mean BMI was 23.9 ± 3.5 kg/m^2^, mean BSA was 1.59 ± 0.15 m^2^, mean sBP was 128 ± 30 mm Hg, and mean dBP was 72 ± 16 mm Hg. Median HR was 85 beats/min, median CI was 2.6 L/min/m^2^, and median eGFR was74.1 mL/min. Of the 215 patients, 12 (5.6%) presented with HF, 26 (12.1%) with MI, 16 (7.4%) with cerebral infarction (excluded cardiogenic), 15 (7.0%) with valvular disease, 24 (11.2%) coronary revascularization (percutaneous coronary intervention or coronary artery bypass grafting), 45 (20.9%) with DM, 92 (42.8%) with HT, and 57 (26.5%) with HL.

**Table 2:** Baseline characteristics in the eciFD and non-eciFD groups.

	Total	eciFD	non-eciFD	
	n = 215	n = 135	n = 80	*P* value
(A) Patient characteristics				
Age	67 (56–76)	71 (63–79)	54 (35–65)	<.0001
Age <61 yrs	74 (34.4%)	23 (17.4%)	51 (63.8%)	<.001
Male	127 (59.1%)	57 (41.0%)	31 (40.8%)	.98
Hight (n = 94)	157.6 ± 8.6	156.3 ± 8.6 (n = 59)	160.0 ± 8.2 (n = 35)	.04
Hight <160 cm	56 (59.6%)	39 (66.1%)	17 (48.6%)	.09
Weight	57.5 (50.4–66.8)	55.0 (49.8–61.0)	65.5 (56.0–72.6)	<.0001
BMI (n = 74)	23.9 ± 3.5	23.6 ± 3.6 (n = 49)	24.5 ± 3.3 (n = 25)	.28
BSA (n = 94)	1.59 ± 0.15	1.56 ± 0.14 (n = 59)	1.63 ± 0.15 (n = 35)	.02
BSA ≤1.6 m^2^	56 (59.6%)	39 (66.1%)	17 (48.6%)	.09
LBM (n = 94)	41.9 ± 4.8	40.9 ± 4.4	43.5 ± 5.0	.01
LBM ≤45 kg	69 (73.4%)	48 (81.4%)	21 (60.0%)	.03
CI	2.6 (2.5–2.8)	2.5 (2.5–2.6)	2.8 (2.6–3.3)	<.0001
CI ≤2.5 L/min/m^2^	86 (40.0%)	71 (52.6%)	15 (18.8%)	<.0001
CO (n = 94)	4.2 (3.8–4.6)	3.9 (3.8–4.3)	4.9 (4.1–6.4)	<.0001
CO ≥4.0 L/min	54 (57.4%)	27 (45.8%)	27 (77.1%)	.003
HR	85 (70–100)	82 (70–101)	88 (70–100)	.11
HR ≤80 bpm	96 (45.1%)	68 (49.3%)	28 (37.3%)	.09
sBP (n = 214)	128 ± 30	132 ± 31	122 ± 29	.03
dBP (n = 199)	72 ± 16	73 ± 16	71 ± 15	.50
HF	12 (5.6%)	7 (5.2%)	5 (6.3%)	.74
MI	26 (12.1%)	17 (12.6%)	9 (11.3%)	.77
Cerebral Infarction (excluded cardiogenic)	16 (7.4%)	12 (8.9%)	4 (5.0%)	.29
Valvular disease	15 (7.0%)	10 (7.4%)	5 (6.3%)	.74
Pulmonary arterial hypertension	2 (0.9%)	1 (0.7%)	1 (1.3%)	.71
Coronary revascularization (PCI or CABG)	24 (11.2%)	16 (11.9%)	8 (10.0%)	.26
DM	45 (20.9%)	29 (21.5%)	16 (20.0%)	.80
HT	92 (42.8%)	63 (46.7%)	29 (36.3%)	.14
HL	57 (26.5%)	44 (32.6%)	13 (16.3%)	.009
Peripheral arterial disease	4 (1.9%)	3 (2.2%)	1 (1.3%)	.61
LC	7 (3.3%)	3 (2.2%)	4 (5.3%)	.27
eGFR	74.1 (55.2–92.5)	67.2 (53.5–86.2)	81.0 (65.3–99.0)	.004
eGFR ≤40 mL/min	18 (8.4%)	13 (9.7%)	5 (6.3%)	.38
Order from EC	167 (77.7%)	104 (77.0%)	63 (78.8%)	.77
(B) contrast factors				
Aortic CTV	339 (287–394)	347 (313–406)	314 (258–374)	.0006
Aortic CTV ≥300 HU	153 (71.1%)	108 (80.0%)	45 (56.3%)	.0002
Left-IV	83 (38.8%)	58 (43.0%)	25 (31.7%)	.10
CM dose mgI/kg	592 (546–624)	602 (574–638)	556 (529–600)	<.0001
CM dose >600 mgI/kg	89 (41.4%)	71 (52.6%)	18 (22.5%)	<.0001
FD	19.7 (18.0–21.1)	20.1 (18.8–21.2)	18.0 (17.0–20.6)	<.0001
FD = 20 ± 1 mgI/kg/sec	71 (33.0%)	59 (43.7%)	12 (15.0%)	<.0001
Injection duration	30.3 (30–30.3)	30.3 (30.3–30.3)	30.3 (29.2–30.3)	.23
BT	92 (42.8%)	59 (43.7%)	33 (41.3%)	.73
Scan delay (n = 138)	35 (33–35)	35 (35–35)	35 (31–35)	.02

BMI = body mass index, BSA = body surface area, BT = bolus tracking, CABG = coronary artery bypass grafting, CI = cardiac index, CM = contrast medium, CO = cardiac output, CTV = computed tomography value, dBP = diastolic blood pressure, DM = diabetes mellitus, eciFD = estimate cardiac index fractional dose, eGFR = estimated glomerular filtration rate, FD = fractional dose, HF = heart failure, HL = hyperlipidemia, HR = heart rate, HT = hypertension, LBM = lean body mass, LC = liver cirrhosis, Left-IV = left upper extremity intra venous injection, MI = myocardial infarction, PCI = percutaneous coronary intervention, sBP = systolic blood pressure.

In terms of items related to contrast-enhanced CT, median aortic CTV was 339 HU, median CM dose was 592 mgI/kg, median FD was 19.7 mgI/kg/sec, median injection duration was 30.3 seconds, and median scan delay was 35 seconds. Of the 215 patients, 92 (42.8%) underwent contrast-enhanced CT with BT. CT was ordered by an EC in 167 patients (77.7%).

### 3.2 Baseline characteristics in eciFD and non-eciFD groups

We compared the baseline characteristics of patients between the eciFD (n = 135) and non-eciFD groups (n = 80). Patients in the eciFD group were significantly older and smaller (lower height, weight, BMI, BSA, and LBM) than those in the non-eciFD group. CI was also significantly lower in the eciFD group.

No significant differences in HR, dBP, HF, MI, cerebral infarction (excluding cardiogenic), DM, HT, liver cirrhosis, valvular disease, pulmonary arterial hypertension, or peripheral arterial disease were seen. The sBP was significantly higher in the eciFD group. Rates of HL were significantly higher, and eGFR was significantly lower in the eciFD group. However, many patients showed no renal dysfunction.

**Figure 6:**
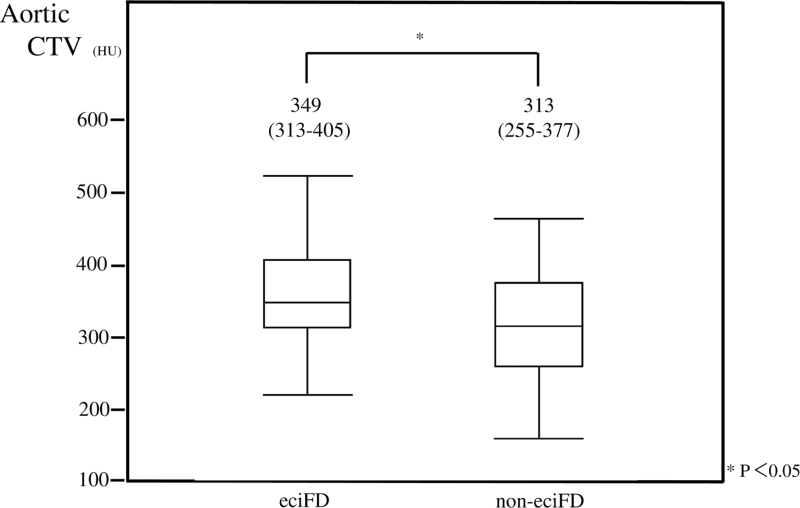
Aortic CTV in the eciFD (n = 135) and non-eciFD (n = 80) groups. The upper end of the box indicates the third quartile and the bottom end indicates the first quartile. The line in the box plot represents the median. The upper whisker indicates the minimum and the lower whisker indicates the minimum. CTV = computed tomography value, eciFD = estimated cardiac index fractional dose.

**Figure 7:**
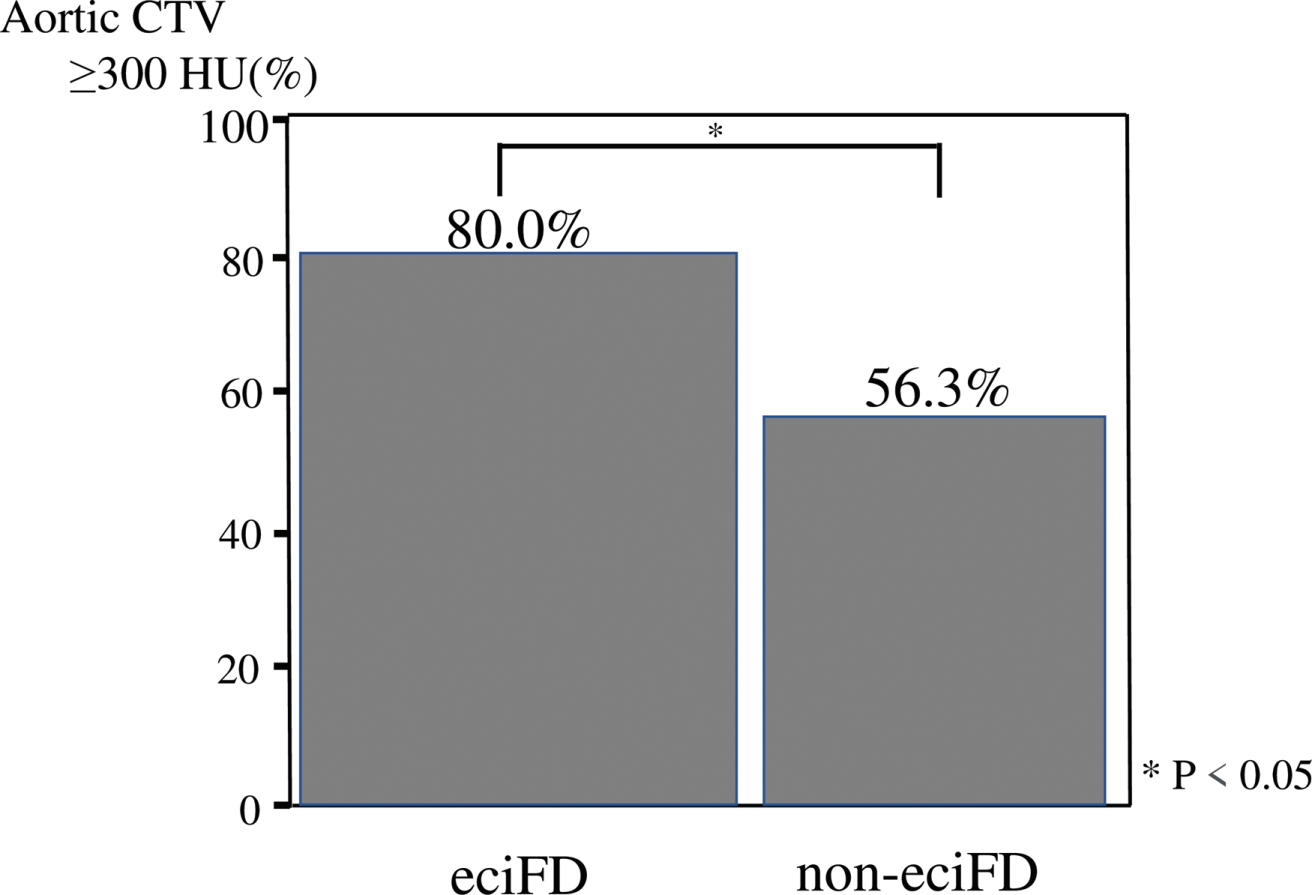
Percentage of patients with aortic CTV ≥300 HU in the eciFD (n = 135) and non-eciFD (n = 80) groups. CTV = computed tomography value, eciFD = estimated cardiac index fractional dose.

With regard to the characteristics of contrast-enhanced CT, aortic CTV (Fig. 6) and the ACTV≥300 (Fig. 7) were significantly higher in the eciFD group. CM dose and FD were significantly higher in the eciFD group, and rates of CM dose >600 mgI/kg and FD = 20 ± 1 mgI/kg/sec were also significantly higher.

No significant differences in injection-site laterality, injection duration, scan delay, or BT rate were seen between groups.

### 3.3 Effect of eciFD on early-phase ACTV≥300

The eciFD protocol was significantly associated with early-phase ACTV≥300 (OR, 3.11; 95%CIs, 1.69–5.73; *P* < .0003), and other significant factors were HR ≤80 beats/min (OR, 2.27; 95%CIs, 1.21–4.3; *P* = .01), BT (OR, 1.87; 95%CIs, 1.0–3.47; *P* = .049), and order from EC (OR, 0.42; 95%CIs, 0.18–0.96; *P* = .04) (Table 3).

**Table 3:** Logistic regression analysis.

	Univariate		Multivariate	
Aortic CTV ≥300 HU predictor	Odds ratio (95%CIs)	*P* value	Odds ratio (95%CIs)	*P* value
Male	0.97 (0.53–1.76)	.91	0.87 (0.45–1.67)	.67
HR ≤ 80 bpm	2.27 (1.21–4.3)	.01	1.93 (0.99–3.78)	.05
eGFR ≤ 40mL/min	0.61 (0.23–1.66)	.34	0.52 (0.17–1.58)	.25
eciFD	3.11 (1.69–5.73)	.0003	3.03 (1.59–5.77)	.001
BT	1.87 (1.0–3.47)	.049	2.02 (1.03–3.95)	.039
Order from EC	0.42 (0.18–0.96)	.04	0.40 (0.17–0.98)	.045
MI	0.74 (0.31–1.75)	.49	0.78 (0.29–2.11)	.63

BT = bolus tracking, CI = cardiac index, CIs = confidence intervals, CTV = computed tomography value, eciFD = estimate cardiac index fractional dose, eGFR = estimated glomerular filtration rate, FD = fractional dose, HR = heart rate, MI = myocardial infarction.

Furthermore, even after adjustment by multiple logistic analysis for 6 factors, including these 3 factors plus the clinically important factors of male sex, eGFR ≤40 mL/min, and history of MI, eciFD remained a significant factor (OR, 3.03; 95%CIs, 1.59–5.77; *P* = .001).

A variance inflation factor >5 was not detected for any models (Table 3), suggesting that variables were free from multicollinearity. No interaction was identified between BT and eciFD (p for interaction = 0.76).

### 3.4 Comparison of eciFD and non-eciFD groups by scan delay determination technique (BT/FIX)

BT and FIX techniques were considered as strong confounders for ACTV≥300 and the utility of eciFD protocol was analyzed by stratification (Tables S1 and S2 Supplemental Digital Content).

**Figure 8:**
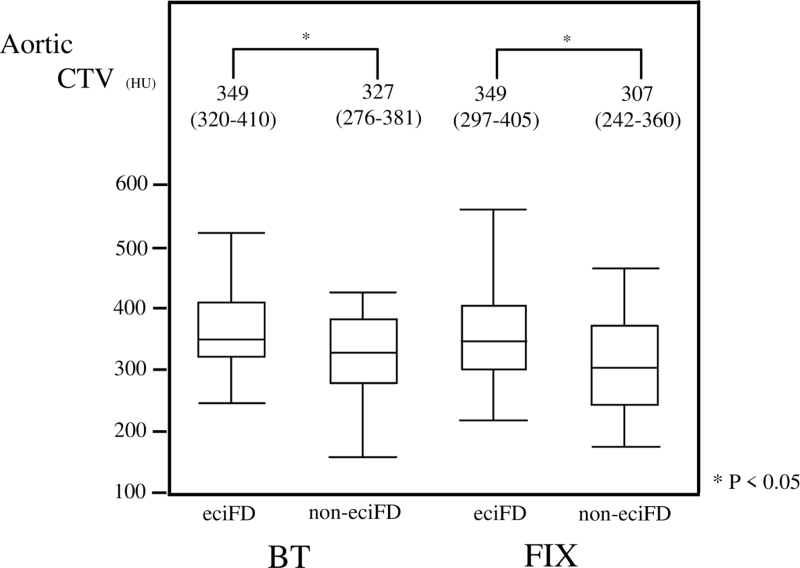
Aortic CTV in the eciFD and non-eciFD groups by scan delay determination technique (BT/FIX). The upper end of the box indicates the third quartile and the bottom end indicates the first quartile. The line in the box plot represents the median. The upper whisker indicates the minimum and the lower whisker indicates the minimum. BT = bolus tracking, CTV = computed tomography value, eciFD = estimated cardiac index fractional dose, FIX = fixed scan delay.

**Figure 9:**
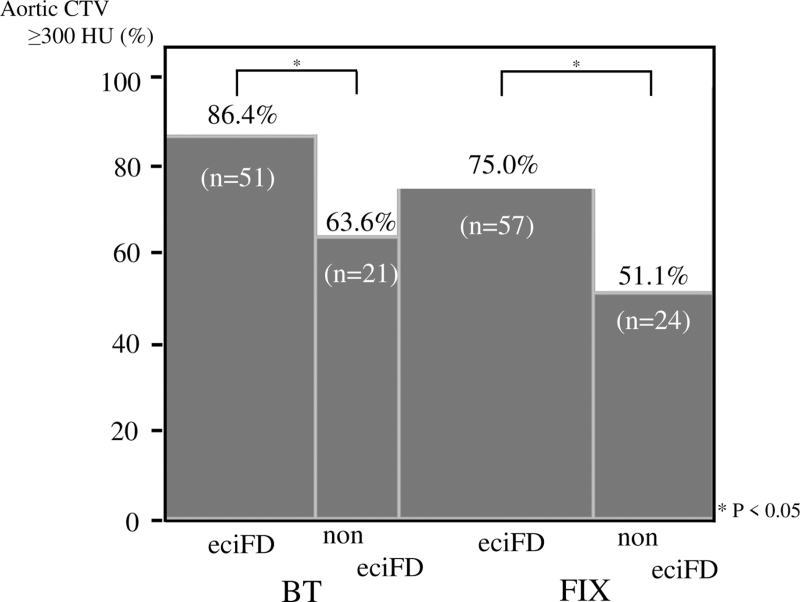
Percentage of patients with aortic CTV ≥300 HU in the eciFD and non-eciFD groups by scan delay determination technique (BT/FIX). BT = bolus tracking, CTV = computed tomography value, eciFD = estimated cardiac index fractional dose, FIX = fixed scan delay.

The eciFD group showed significantly lower weight and lower CI and eGFR, and a significantly greater frequency of HL compared to the non-eciFD group with both FIX and BT, while sBP was higher only with BT. With regard to the characteristics of contrast-enhanced CT, levels of aortic CTV (Fig. 8) and %aortic ACTV≥300 (Fig. 9) in the eciFD group were high with both FIX and BT.

In terms of CM dose and percentage of CM dose >600 mgI/kg and FD = 20 ± 1 mgI/kg/sec, the eciFD group showed significantly higher values for both FIX and BT. Scan delay was significantly higher only with BT.

With both FIX and BT, the eciFD protocol was a significant factor for early-phase ACTV≥300, eciFD protocol with FIX (OR, 2.61; 95%CIs, 1.21–5.64; *P* = .02), and eciFD protocol with BT (OR, 3.64; 95%CIs, 1.3–10.2; *P* = .01). Other significant factors were HR ≤80 beats/min (OR, 2.94; 95%CIs, 1.3–6.6; *P* = .01) with FIX. After adjusting for several confounders by multiple logistic regression analysis, the eciFD protocol remained a significant factor for both FIX (OR, 2.32; 95%CIs, 1.03–5.26; *P* = .04) and BT (OR, 3.76; 95%CIs, 1.25–11.2; *P* = .02).

### 3.5 Aortic CTV and ACTV≥300 in eciFD and non-eciFD groups by scan delay determination technique (BT/FIX)

With aortic CTV (Fig. 8) and ACTV≥300 (Fig. 9), frequency of the eciFD protocol was significantly higher for both FIX and BT. In particular, the combination of eciFD protocol and BT showed the highest ACTV≥300 (86.4%).

## 4 Discussion

This study revealed 2 main points. First, introduction of the eciFD protocol allowed higher aortic CTV to be obtained more stably than with the conventional method. Second, the eciFD protocol with the BT obtained the best aortic CTV among the various combinations examined.

### 4.1 Effect of the eciFD protocol on aortic CTV

Introduction of the eciFD protocol was found to more stably provide higher aortic CTV than the conventional method. The FD on which the eciFD protocol was based can be expressed as the amount of iodine per unit body weight per unit time. When the amount of iodine per unit body weight and injection duration are constant and FD is constant, a stable TEC can be obtained according to the physique, and a target imaging effect can be obtained.^[5,8,9,12]^ This is consistent with the report by Awai and Hiraishi^[13]^ that aortic peak time and enhancement were closely related to injection duration when contrast volume and protocol are determined by patient weight. However, even when the amount of iodine per unit body weight and injection duration are constant and FD is constant, TEC fluctuates due to the influence of the circulatory dynamics for the individual, as a factor that impairs the reproducibility of aortic contrast enhancement in early-phase dynamic CT.^[5]^

In this regard, the eciFD protocol is a clear, simple protocol in which CI is estimated according to the age, and FD according to CI is administered with a fixed amount of iodine per body weight and injection duration. Administration of CM per unit body weight in consideration of hemodynamics improves and stabilizes contrast enhancement of the aorta on dynamic CT.

### 4.2 Protocol for eciFD with BT

The eciFD protocol with BT obtained the best aortic CTV of the various combinations.

BT offers the advantage that the arrival time of CM is easily observed in each patient and scan delay can be determined according to the hemodynamics, but shows the disadvantage that an appropriate peak aortic TEC cannot be guaranteed.^[14–17]^

The eciFD protocol facilitates the formation of TEC with peaks of 300 to 400 HU. This seems to reinforce the drawback of the BT technique that the aortic TEC peak cannot be guaranteed. In addition, aortic contrast enhancement was considered to be achieved by appropriately determining the arrival time of CM using BT.

Thus, the eciFD protocol, which produces a consistent and similar contrast effect for all patients, may help radiologists reach diagnoses.

CI as an index for estimating FD in this study was expressed by a formula obtained by dividing CO by BSA, and thus represented a factor that takes into account body size to determine CO.

CO strongly affected contrast enhancement of the aorta,^[18]^ and BSA was reported by Masuda et al^[19]^ as the strongest factor in a regression analysis study of lower extremity vessels. CI consisting of these factors is an important factor for aortic contrast enhancement and is useful as an indicator for determining FD.

### 4.3 Clinical implications

Several references related to FD-based contrast protocols for coronary and hepatic multiphasic CT have been reported.^[20,21]^ However, the eciFD protocol can be applied over a wide radiographic range from neck to pelvis, including EC cases. In addition, this protocol is easily used in EC, because the dosage can be determined individually for each case. In the elderly, high aortic CTV was obtained even with low FD, long injection duration, and low injection rate using the eciFD protocol. The risk of CM extravasation may thus be reduced. Many reports have clarified that low tube voltage imaging and dual-energy imaging,^[22–26]^ and introduction of a spiral flow-generating tube,^[27,28]^ can reduce the amount of CM needed. This eciFD protocol can provide a basis for reducing CM while maintaining contrast enhancement of the aorta.

## 5 Limitations

Some limitations need to be considered for this study. First, this study did not randomly assign the eciFD protocol, and as a prospective observational study, selection biases and confounders may not have been controlled for. In fact, the eciFD protocol could not be applied in 80 of 215 patients (37.2%). Similarly, the protocol was not well understood by the night staff in some cases, and body weight was unknown at the time of scanning due to the large number of EC patients, and in fact the dose of CM was somewhat lower or higher than optimal.

Second, we recommended the use of the tables shown in Figures 4 and 5 to guide administration in this study. However, we still allowed the automated injector to calculate the injection parameters. In such cases, the injection rate and CM volume were set by inputting the FD and patient weight indicated by the eciFD protocol into the automated injector, but the injection duration was set as 30 seconds. Therefore, the dose of CM unexpectedly exceeded 600 mgI/kg in some cases.

Finally, only single-stage injection of CM was considered in this study. The FD of the eciFD protocol is likely to change with the use of saline flushes^[29,30]^ and the CM dose may be reduced.^[2,10]^

## 6 Conclusion

This eciFD protocol proved useful for improving contrast enhancement of the aorta regardless of the technique for determining scan delay. In particular, the protocol was effective for reinforcing BT and obtaining optimal contrast enhancement of the aorta. Further study using a randomized controlled study is warranted.

## Acknowledgments

We would like to thank Dr Jun Nakazato for his advice in the cardiovascular field, Dr Wataru Higashiura for his advice in the field of radiology and for reviewing the manuscript, and FORTE (www.forte-science.co.jp) for English language editing.

## Author contributions

**Conceptualization:** Tadashi Kuba

**Formal analysis:** Sadayuki Murayama

**Supervision:** Shinichiro Ueda

**Writing – original draft:** Tadashi Kuba

**Writing – review & editing:** Akihiro Tokushige

## Supplementary Material

Supplemental Digital Content

## Supplementary Material

Supplemental Digital Content
