# Supplementary material for: Proposal of a novel protocol using estimated cardiac index fractional dose to improve aortic contrast enhancement for early-phase dynamic CT
Source: Medicine (Baltimore). 2022 Jun 24;101(25):e29410. doi: 10.1097/MD.0000000000029410 (PMC9276326; doi:10.1097/MD.0000000000029410)
Supplement: Supplemental Digital Content [file medi-101-e29410-s001.docx]

**Supplementary Table 1**

**Baseline characteristics of eciFD vs non-eciFD** **by scan delay time determination technique (BT /FIX)**

|  | FIX  n=123 | | Pvalue | BT  n=92 | | Pvalue |
| --- | --- | --- | --- | --- | --- | --- |
|  | eciFD  n=77 | Non-eciFD  n=46 |  | eciFD  n=59 | Non-eciFD  n=33 |  |
| **(A)**  **Patient characteristics** |  |  |  |  |  |  |
| Age | 68  (63-77) | 53  (36-68) | <0.0001 | 72  (63-81) | 50  (20-60) | <0.0001 |
| Age  <61 y  (n=74) | 11  (14.3 %) | 28  (60.9 %) | <0.0001 | 9  (15.3 %) | 26  (78.8 %) | <0.0001 |
| Male | 44  (57.1 %) | 27  (58.7 %) | 0.87 | 36  (61.0 %) | 20  (60.6 %) | 0.97 |
| Hight  (n=94) | 157.2±8.3 | 158.6±7.9 | 0.56 | 154.9±9.3 | 160.9±8.4 | 0.04 |
| Hight  <160 cm  (n=56) | 22  (64.7 %) | 10  (55.6 %) | 0.21 | 16  (69.6 %) | 8  (42.1 %) | 0.07 |
| Weight  (n=215) | 56.5  (51.0-62.0) | 60.5  (51.2-72.9) | 0.04 | 54.0  (47.0-61.2) | 65.0  (57.8-71.0) | <0.0001 |
| BMI  (n=74) | 23.6±3.9 | 24.6±3.6 | 0.37 | 23.5±3.0 | 24.3±2.8 | 0.53 |
| BSA  (n=94) | 1.58±0.12 | 1.63±0.17 | 0.17 | 1.53±0.03 | 1.63±0.13 | 0.05 |
| BSA  ≤ 1.6 m²  (n=56) | 23  (67.7 %) | 11  (61.1 %) | 0.64 | 14  (60.9 %) | 8  (42.1 %) | 0.23 |
| LBM  (n=94) | 40.7  (39.0-43.7) | 41.8  (39.1-46.6) | 0.34 | 38.8  (35.7-45.0) | 44.6  (39.3-46.7) | 0.03 |
| LBM ≤ 45 kg  (n=69) | 29  (85.3 %) | 11  (61.1 %) | 0.049 | 17  (73.9 %) | 12  (63.2 %) | 0.45 |
| CI  (n=215) | 2.6  (2.5-2.6) | 2.9  (2.6-3.3) | <0.0001 | 2.5  (2.5-2.6) | 2.9  (2.6-3.9) | <0.0001 |
| CI  ≤ 2.5 L/min/m²  (n=86) | 35　(45.5 %) | 9  (19.6 %) | 0.004 | 37  (62.7 %) | 5  (15.2 %) | <0.0001 |
| CO  (n=52) | 3.96  (3.81-4.26) | 4.77  (3.96-6.26) | 0.08 | 3.8  (3.5-4.4) | 4.9  (4.5-6.8) | <0.0001 |
| CO  ≥ 4.0 L / min  (n=54) | 16  (47.1 %) | 13  (72.2 %) | 0.08 | 9  (39.1 %) | 16  (84.2 %) | 0.003 |
| HR  (n=213) | 84  (72-100) | 93  (69-104) | 0.51 | 81  (67-103) | 82  (71-97) | 0.58 |
| HR ≤ 80 bpm  (n=96) | 37  (48.1 %) | 16  (35.6 %) | 0.18 | 29  (50.0 %) | 14  (42.4 %) | 0.49 |
| sBP  (n=214) | 128±30 | 128±31 | 0.91 | 135±32 | 116±27 | 0.005 |
| dBP  (n=199) | 72±15 | 74±16 | 0.53 | 73±17 | 68±13 | 0.15 |
| HF  (n=12) | 2  (2.6 %) | 2  (4.4 %) | 0.60 | 5  (8.5 %) | 3  (9.1 %) | 0.92 |
| MI  (n=26) | 10  (13 %) | 4  (8.7 %) | 0.46 | 8  (13.6 %) | 4  (12.1 %) | 0.84 |
| Cerebral Infarction (excluded cardiogenic)  (n=16) | 7  (9.1 %) | 3  (6.5 %) | 0.61 | 4  (6.8 %) | 2  (6.1 %) | 0.89 |
| Valvular  Disease  (n=15) | 5  (6.5 %) | 2  (4.4 %) | 0.62 | 6  (10.2 %) | 2  (6.1 %) | 0.50 |
| Pulmonary Arterial  Hypertension  (n=2) | 1  (1.3 %) | 1  (2.2 %) | 0.71 | 0  (0 %) | 0  (0 %) | 0 |
| Coronary Revascularization  (PCI or CABG)  (n=24) | 10  (13.0 %) | 3  (6.5 %) | 0.26 | 7  (11.9 %) | 4  (12.1 %) | 0.97 |
| DM  (n=45) | 17  (22.1 %) | 9  (19.6 %) | 0.74 | 14  (23.7 %) | 5  (15.2 %) | 0.33 |
| HT  (n=92) | 39  (50.7 %) | 16  (34.8 %) | 0.09 | 27  (45.8 %) | 10  (30.3 %) | 0.15 |
| HL  (n=57) | 26  (33.8 %) | 7  (15.2 %) | 0.02 | 20  (33.9 %) | 4  (12.1 %) | 0.02 |
| Pripheral  Arterial Disease  (n=4) | 2  (2.6 %) | 0  (0 %) | 0.27 | 1  (1.7 %) | 1  (3.0 %) | 0.67 |
| LC  (n=7) | 0  (0 %) | 2  (4.4 %) | 0.07 | 3  (5.1 %) | 2  (6.1 %) | 0.84 |
| eGFR  (n=214) | 67.9  (52.8-88.6) | 88.5  (68.1-102.3) | 0.002 | 65.0  (53.2-80.2) | 82.5  (60.4-105.9) | 0.013 |
| eGFR  ≤ 40 mL/min  (n=18) | 8  (10.4 %) | 1  (2.2 %) | 0.09 | 5  (8.6 %) | 4  (12.1 %) | 0.59 |
| Order from EC  (n=167) | 54  (70.1 %) | 36  (78.3 %) | 0.97 | 49  (83.1 %) | 28  (84.9 %) | 0.82 |
| **(B)contrast factors** |  |  |  |  |  |  |
| aortic CTV | 349  (297-405) | 307  (242-360) | 0.003 | 349  (320-410) | 327  (276-381) | 0.007 |
| aortic CTV  ≥ 300 HU  (n=153) | 57  (75.0 %) | 24  (51.1 %) | 0.01 | 51  (86.4 %) | 21  (63.6 %) | 0.01 |
| Left-IV  (n=83) | 38  (49.4 %) | 16  (35.6 %) | 0.14 | 19  (32.2 %) | 10  (30.3 %) | 0.85 |
| CM dose  mgI/kg | 605.3±43.6 | 569.5±51.4 | <.0001 | 592.5±49.0 | 571.1±39.4 | 0.03 |
| CM dose  ＞600 mgI/kg  (n=89) | 42  (54.6 %) | 12  (26.1 %) | 0.002 | 28  (47.5 %) | 7  (21.2 %) | 0.01 |
| FD | 19.9±1.7 | 19.3±3.8 | 0.34 | 20.0±1.7 | 19.4±3.1 | 0.34 |
| FD=20±1  mgI/kg/sec | 29  (37.7 %) | 7  (15.2 %) | 0.008 | 28  (47.5 %) | 7  (21.2 %) | 0.01 |

| Scan delay  (n=138) | 35  (35-35) | 35  (35-35) | 0.21 | 35  (32-40) | 32  (30-35) | 0.003 |
| --- | --- | --- | --- | --- | --- | --- |
| Injection  Duration | 30.3  (30.3-30.3) | 30.3  (29.3-30.3) | 0.07 | 30.3  (30-30.3) | 30.3  (28.6-30.3) | 0.57 |

eciFD=estimate cardiac index Fractional Dose, BMI=Body Mass Index, BSA=Body Surface Area, LBM=Lean Body Mass, CI=Cardiac Index, CO=Cardiac Output, HR=Heart Rate, dBP=diastolic Blood Pressure, sBP= systolic blood pressure, HF=Heart Failure, MI= Myocardial Infarction, DM= Diabetes Mellitus, HT= Hyper Tension, HL= Hyper Lipidemia, LC= Liver cirrhosis, eGFR=estimated Glomerular Filtration Rate, aortic CTV=aortic Computed Tomography Value, Left-IV=Left upper extremity Intra Venous injection, CM dose=Contrast Medium dose, FD=Fractional Dose, BT=Bolus Tracking
