# Supplementary material for: Proposal of a novel protocol using estimated cardiac index fractional dose to improve aortic contrast enhancement for early-phase dynamic CT
Source: Medicine (Baltimore). 2022 Jun 24;101(25):e29410. doi: 10.1097/MD.0000000000029410 (PMC9276326; doi:10.1097/MD.0000000000029410)
Supplement: Supplemental Digital Content [file medi-101-e29410-s002.docx]

**Supplementary Table 2**

**Logistic regression analysis by scan delay determination technique (BT /FIX)**

|  | Univariate | | | | Multivariate | | | |
| --- | --- | --- | --- | --- | --- | --- | --- | --- |
| aortic CTV  ≥ 300 HU  predictor | FIX | Pvalue | BT | Pvalue | FIX | Pvalue | BT | Pvalue |
|  | OR  (95%CI) |  | OR  (95%CI) |  | OR  (95%CI) |  | OR  (95%CI) |  |
| eciFD | 2.61  (1.21-5.64) | 0.02 | 3.64  (1.3-10.2) | 0.01 | 2.32  (1.03-5.26) | 0.04 | 3.76  (1.25-11.2) | 0.02 |
| Male | 1.39  (0.66-2.95) | 0.39 | 0.44  (0.14-1.34) | 0.15 | 1.18  (0.53-2.65) | 0.69 | 0.42  (0.13-1.44) | 0.17 |
| HR  ≤ 80 bpm | 2.94  (1.3-6.6) | 0.01 | 1.46  (0.53-4.0) | 0.46 | 2.40  (1.0-5.73) | 0.05 | 1.50  (0.49-4.54) | 0.47 |
| eGFR  ≤ 40 mL/min | 1.0  (0.25-4.38) | 0.96 | 0.30  (0.07-1.26) | 0.10 | 0.92  (0.19-4.51) | 0.92 | 0.43  (0.08-2.26) | 0.32 |
| Order  from EC | 0.42  (0.17-1.08) | 0.07 | 0.22  (0.03-1.77) | 0.15 | 0.60  (0.22-1.65) | 0.33 | 0.20  (0.02-1.74) | 0.14 |
| MI | 0.93  (0.29-2.96) | 0.90 | 0.5  0.5  (0.13-1.87) | 0.30  0.30 | 0.79  0.79  (0.22-2.87) | 0.79  0.79 | 0.80  (0.17-3.87) | 0  0.78 |

eciFD=estimated cardiac index Fractional Dose, CI=Cardiac Index, HR=Heart Rate, HF=Heart Failure, eGFR=estimated Glomerular Filtration Rate, CTV=Computed Tomography Value, CM=Contrast Medium, FD=Fractional Dose, BT=Bolus Tracking,
